# Supplementary material for: Improving HIV testing and retention among adolescents and youths: Lessons from a quasi-experimental study of the Red-Carpet Program in Malawi
Source: PLOS Glob Public Health. 2024 Dec 19;4(12):e0004072. doi: 10.1371/journal.pgph.0004072 (PMC11658608; doi:10.1371/journal.pgph.0004072)
Supplement: Attachment — Submitted filename: PLOS_rev_8Oct24.pdf [file pgph.0004072.s005.pdf]

Kind Madam/Sir,

I hope this message finds you well. I have thoroughly reviewed your article titled " Improving HIV Testing and Retention among Adolescents and Youth: Lessons from the quasi-experimental study of the Red-Carpet Program in Malawi" and wanted to share my feedback with you.

Strengths: The article is well written. The outcome of the study is to assess the effect of RCP on Provider-Initiated HIV testing, linkage to care, and retention in care among AYLHIV which is an advancement in the management of HIV diagnosed people. This could help in identifying appropriate actions and interventions in improving the HIV testing, linkage to care and retention in care.

Suggestions: As the baseline retention rates are matched between the interventional and non-interventional sites, in the outcomes you could add ‘Change in retention rates’. When abbreviations are used, please use capitals for the full form.
Results: Percentages are wrong. You have excluded the ‘missed’ variable while calculating the percentages

Conclusion:

Overall, your article presents a valuable contribution to HIV management. The thorough analysis and the innovative approach to methodology are commendable. Your insights have the potential to significantly influence future research in this area.

Thank you once again for the opportunity to review your work. I look forward to seeing how you refine and develop the article.

Best regards
