# Supplementary material for: Improving HIV testing and retention among adolescents and youths: Lessons from a quasi-experimental study of the Red-Carpet Program in Malawi
Source: PLOS Glob Public Health. 2024 Dec 19;4(12):e0004072. doi: 10.1371/journal.pgph.0004072 (PMC11658608; doi:10.1371/journal.pgph.0004072)
Supplement: Attachment — Submitted filename: Rebuttal letter_6_Nov_24.pdf [file pgph.0004072.s006.pdf]

Dear Editor,

We appreciate your valuable feedback and the opportunity to address the comments from the reviewers. Please find below a point-by-point response to the queries raised.

PLOS Review feedback

Comment 1:I hope this message finds you well. I have thoroughly reviewed your article titled " Improving HIV Testing and Retention among Adolescents and Youth: Lessons from the quasi experimental study of the Red-Carpet Program in Malawi" and wanted to share my feedback with you. Strengths: The article is well written. The outcome of the study is to assess the eNect of RCP on Provider-Initiated HIV testing, linkage to care, and retention in care among AYLHIV which is an advancement in the management of HIV diagnosed people. This could help in identifying appropriate actions and interventions in improving the HIV testing, linkage to care and retention in care.
Response to Comment 1: Thank you for your feedback

Query 1: Suggestions: As the baseline retention rates are matched between the interventional and non-interventional sites, in the outcomes you could add ‘Change in retention rates’.

Response 1: Thank you for your suggestion. We appreciate your feedback. However, we would not suggest that we describe it as comparing the change in retention as the focus was not a pre and post study.i.e we did not focus on calculating retention before and after the intervention across sites. Rather, as we support more than 30 facilities in Blantyre which have a variation in retention rates in adolescents, we used the baseline retention rates to select sites who were at least similar in retention before the intervention. We focused at comparing outcomes in the non-intervention and intervention sites after the intervention.

Query 2: When abbreviations are used, please use capitals for the full form.

Response 2:We have updated all abbreviations and have used capital letters across the document.

Query 3: Results: Percentages are wrong. You have excluded the ‘missed’ variable while calculating the percentages

Response 3: We have amended the table and removed that line to avoid confusion. We have added a note under the table. For the missing variables, there was no valid response provided and we propose to not include them in the denominator. We have done that for the variables on HIV testing and viral load results.

Comment 2: Conclusion: Overall, your article presents a valuable contribution to HIV management. The thorough analysis and the innovative approach to

methodology are commendable. Your insights have the potential to significantly influence future research in this area.Thank you once again for the opportunity to review your work. I look forward to seeing how you refine and develop the article.
Response to Comment 2: Thank you for your feedback

Sincerely,

Rachel Chamanga Kanyenda
