# Supplementary material for: Improving HIV testing and retention among adolescents and youths: Lessons from a quasi-experimental study of the Red-Carpet Program in Malawi
Source: PLOS Glob Public Health. 2024 Dec 19;4(12):e0004072. doi: 10.1371/journal.pgph.0004072 (PMC11658608; doi:10.1371/journal.pgph.0004072)
Supplement: Attachment — Submitted filename: Manuscript Coverletter Apr 2024.doc [file pgph.0004072.s002.doc]

Rachel Chamanga Kanyenda
P.O. Box 2543

Lilongwe, Malawi

03 Apr 24

The Editors-In-Chief

PLOS Global Public Health

1265 Battery Street, Suite 200
San Francisco, CA 94111
United States

Dear Editors-In-Chief,

We are pleased to re-submit to PLOS Global Public Health the research article entitled, **“Improving HIV Testing and Retention among Adolescents: Lessons from the Red-Carpet Program in Malawi**”

This quasi-experimental study utilized secondary data that was collected under the “Evaluation of Outcomes achieved through integrated HIV/AIDS and TB Prevention, Care and Treatment Programs in Malawi” (an umbrella protocol) in order to evaluate an intervention called the Red Carpet Program which was provided to newly-initiated HIV positive adolescents on ART . We have completed the PLOS Human Participant Checklist and have attached the Institutional Review Board approvals for this study.

Please note that I have attached the following:

1. Initial approval of the study protocol dated 12 Sep 2018 which details the Ministry of Health (MOH) registers that can be used including the HTC and ART register
2. Continual approval of the study of the years 2019-2020; 2020-2021; 2021-2022;2022-2023 and 2023-2024
3. Approval of an amendment dated 29 Nov 2019 where there was a Change in the Principal Investigator (PI)
4. Approval of an amendment dated 25 Mar 2022 where use of individual nationwide data was made including use of electronic medical records
5. The study protocol dated 09 Mar 2022.

We look forward to your feedback on this manuscript

Sincerely,

Dr Rachel Chamanga Kanyenda
