# Supplementary material for: Improving HIV testing and retention among adolescents and youths: Lessons from a quasi-experimental study of the Red-Carpet Program in Malawi
Source: PLOS Glob Public Health. 2024 Dec 19;4(12):e0004072. doi: 10.1371/journal.pgph.0004072 (PMC11658608; doi:10.1371/journal.pgph.0004072)
Supplement: Attachment — Submitted filename: PLOS_ review.docx [file pgph.0004072.s003.docx]

**Review of the study :**

**Title: Improving HIV Testing and Retention among Adolescents: Lessons from the Red- Carpet Program in Malawi**

**Title:**

Design of the study not mentioned

**Abstract:**

Methodology: Its mentioned retention rates were matched between the interventional and non-interventional sites. But isn’t looking at the retention rates among patients an outcome of the study. Outcomes cannot be matched

**Methodology:**

How can the outcome (retention in care) be matched

Inclusion and exclusion criteria of participants from interventional and non interventional sites

Sample size calculation not mentioned

**Results**

Table 1 : percentages are wrong. You have excluded the ‘missed’ variable while calculating the percentages

Previous HIV test done participants are also included. But you have mentioned in methodology only newly diagnosed HIV patients are included in the study. It could be a bias in the study.

Kindly make the changes.
