# Supplementary material for: Improving HIV testing and retention among adolescents and youths: Lessons from a quasi-experimental study of the Red-Carpet Program in Malawi
Source: PLOS Glob Public Health. 2024 Dec 19;4(12):e0004072. doi: 10.1371/journal.pgph.0004072 (PMC11658608; doi:10.1371/journal.pgph.0004072)
Supplement: Attachment — Submitted filename: Rebuttal letter_26_July_24.docx [file pgph.0004072.s004.docx]

Dear Editor,

We appreciate your valuable feedback and the opportunity to address the comments from the reviewers. Please find below a point-by-point response to the queries raised.

**PLOS Review feedback**

**Title:**

**Query 1:** Design of the study not mentioned

**Response 1**: The study design which was a quasi-experimental study has been added to the title

**Abstract:**

**Query 2:** Methodology: Its mentioned retention rates were matched between the interventional and non-interventional sites. But isn’t looking at the retention rates among patients an outcome of the study. Outcomes cannot be matched

**Response 2:** Please note that these were baseline retention rates before the intervention was deployed. We matched on retention because we did not want to have sites that had varying retention amongst the adolescents before the intervention. We wanted sites that had similar retention rates before the intervention to ensure that the other sites were not already performing better compared to others; that way we could somehow be able to attribute changes in retention after implementation of the intervention to the program. We have added this from line 115 to line 121 of the methodology section

**Methodology:**

**Query 3**: How can the outcome (retention in care) be matched

**Response 3:** As explained we, we wanted sites that had similar retention rates before the intervention to ensure that the other sites were not already performing better compared to others; that way we could somehow be able to attribute changes in retention after implementation of the intervention to the program

**Query 4:** Inclusion and exclusion criteria of participants from interventional and non interventional sites

**Response 4:** The inclusion and exclusion criteria has been clarified in more details from line 138 to line 145. At the intervention sites, we included all diagnosed adolescents and youths living with HIV (AYLHIV) that were newly initiated on antiretroviral therapy (ART) and exposed to the intervention through registration in the Red-Carpet enrolment register; we excluded newly initiated AYLHIV on ART who had not been exposed to the Red Carpet intervention or those newly initiated AYLHIV on ART who had transfer-into the facility . At the non-intervention sites, all newly diagnosed AYLHIV initiated on ART, as documented in the ART registry during the same period, were included in the study; we excluded newly diagnosed AYLHIV who had transferred into the facility

**Query 5:**Sample size calculation not mentioned

**Response 5:** We included all AYLHIV that meet the inclusion criteria and thus sample size calculation was not deemed necessary. However, we have revised that section on the inclusion and exclusion criteria to clearly outline this.

**Results**

**Query 6:** Table 1 : percentages are wrong. You have excluded the ‘missed’ variable while calculating the percentages

**Response 6**: We believe that the calculation of the proportions is correct. The variables shown with proportions are the ones that were available for analysis and came out in the STATA output. The “missed” variable have no data at all and do not show up in the STATA output.

**Query 7:** Previous HIV test done participants are also included. But you have mentioned in methodology only newly diagnosed HIV patients are included in the study. It could be a bias in the study.

**Response 7**: We included only adolescents and youth newly-initiated on ART. The ones that had previously tested positive had not ever started on ART. If they had previously tested positive and had started ART elsewhere, they are considered as transfer ins. The transfer-ins were excluded from the study**.** We have amended the wording throughout the document to emphasize on the newly-initiated adolescents and youth on ART.

Kindly make the changes.

Editor`s comments

EDITOR:

Overall the manuscript is well written. However, I request you to kindly address the following-

**Query 8:**1. The manuscript refers to both adolescents and young adults, hence please consider adding ' in the title.

**Response 8**: That addition has been made to the title

**Query 9:** 2. The word 'impact' is used in the last paragraph of the introduction. Kindly replace with effect as impact is a long term effect and this study assesses outcomes at 6 and 12 months post ART initiation.

**Response 9:** That correction has been made. Please see line 104

**Query 10:** Kindly clarify if all the AYLHIV in records at the intervention and nonintervention sites were included. If all were included, a sample size calculation is not necessary.

**Response 10:** All the AYLHIV who met the inclusion criteria were included in the study and hence a sample size calculation was not necessary. This has been highlighted from line 138 to line 145

**Query 11:**3. It is unclear what 'baseline retention rates' in the inclusion of sites means.

**Response 11:** The baseline retention is the retention rate of the adolescents in the various facilities prior to implementation of the programs. It is like a bench mark.We wanted sites which had similar retention rates prior to the intervention in order to avoid bias**.**

**Query 12:** Also non-intervention sites were selected based on numbers of adolescents in care and retention rates at would it not affect the outcome studied? Selecting sites based on similar rates of diagnosis would be more appropriate.

**Response 12:** This is well noted. We did considered rates of diagnosis ( case identification) among AYLHIV amongst other criteria in our matching. We did match on sites with similar rates of newly diagnosed AYLHIV. We also matched on that the sites were urban primary health care facilities; and had similar adolescent patient volumes in care. Lastly, we also matched on baseline retention in order to limit the time-varying characteristics of retention due to other factors**.** The baseline retention, is the retention rate prior to the intervention. We did not want to have facilities which had variations in the retention rate prior to the intervention as it would create some bias.

**Query 13:** 4. Were all AYLHIV during the study period at each site included? If not what were the exclusion criteria e.g., incomplete records, those diagnosed at the facility but belonging to a different region.

**Response 13:** We included all adolescents who met the inclusion criteria. We excluded adolescents who had been on ART before or those that had been diagnosed elsewhere and where transferred to the facility**.**This has been explained on line 138 to line 145

**Query 14:**5. Were AYLHIV who missed the last visit </=2 months ago included in alive and active in care? Usually what is the practice regarding dispensing medications in Malawi?- is this monthly or once in 2 months?

**Response 14:** At the time when we were implementing this evaluation this was around the COVID-19 pandemic where multi-month dispensing had been rolled out. Adolescents and young people living with HIV were provided one monthly refills; then 3 monthly refills; then afterwards six-monthly ART refills. This was different from the pre-COVID-19 era when they were receiving one-month refills. However, it also depended on a case by case basis as some adolescents needed close follow up and were on monthly refills.

If the patient missed the last visit for less than 2 months with the expectation that they had run out of drugs for < 2 months they were considered as not defaulted yet. Those scenarios are considered as missed visits. Please see extract from the Malawi National Guidelines

**Query 15:**6. Is there a better term for Defaulted? Health programmes tend to use the term lost-to-follow-up instead of default. Kindly consider changing this- unless of course the National Program in Malawi uses the term 'default'. Also, does default include AYLHIV who returned to care with more than 2 months of missing follow-ups?

**Response 15:** The National Program synonymously (interchangeably) uses the terminology of defaulters or loss to follow up. The Electronic Medical Records where we accessed this data uses the terminology of default.

If one returns into care within 2 months of missing follow up they are considered as alive and in care. In this study adolescents who may have missed > 2 months of study visit or ART drugs , but came back to care before the one year mark, were considered alive on ART. For this study, we prefer to use the word defaulting from care.

**Query 16:**7. Please indicate the total person-time follow-up in the study and in the intervention and non-intervention arms. Also, would be good to have mean/median time to treatment initiation.

**Response 16:** The total person time for follow-up in the study was 627.02 person-days. The total follow up time in the intervention sites is 417.14 person-days and the total follow up time in the non-intervention 209.88. We have added this on line 181 t 182. The mean time to ART initiation was 0.19 days in intervention sites and 0.25 days in non-intervention sites. We have added this on line 191 to line 192

**Query 17:**8. Kindly discuss limitations of the methodology in a bit more detail- the quasi - experimental design, the selection of non-intervention sites, quality of records and the data at all sites to name a few. On the other hand, small sample size is generally not included under limitations, especially, if all records were included. Reasons why all records were not included could be a limitation.

**Response 17:** Thank you for this comment. We have updated the discussion section with additional limitations. Please see line 344 to line 347

**Query 18:** 9. Minor language edits necessary e.g., "AYLHIV patients", 63.1% of the "patients with AYLHIV" and "the overall retention rates at 12 months after post-ART".  Also kindly use standard teminilogy through-out the document,

**Response 18:** We have made all corrections to the mentioned errors and have used standard terminology through-out the document.

Reviewer 1

**Comment 1:**Reviewer #1: While their research methodology is robust and their findings significant, the article requires minor corrections. Overall, this study serves as a crucial contribution to the field, highlighting the urgent need for action in addressing the strategies to improve HIV screening and retention in care to achieve the 95*95*95 targget by 2025. Thank you for the opportunity to review this enlightening article.

**Response to comment**: Thank you for your comment.

Reviewer #2: At the outset, I must congratulate the authors of the study and the EGPAF team at Malawi for the wonderful work you all are doing at the helm of the HIV epidemic. I must definitely appreciate the efforts taking in improving the AYLHIV outcomes, a very important subgroup of the PLHIV who require extra care and effort which the RCP is an embodiment of.

Strengths of the Study:
1. Well structured study with good methodological explanation.
2. An important separation with use of two groups for Analysis based on the transferred out individuals which could act as a large confounding group in evaluation of your study.
3. Intelligently written and well presented study.

Suggestions:
I had a few semantic errors which I have brought to your notice:

**Query 19:**Line 52 - Would suggest use of p =0.012, instead of just 0.012.

**Response 19:** This has been corrected. Please see line 52

**Query 20:**Line 148 - Would suggest use of d) for the 4th treatment outcome " transferred out"

**Response 20:** This has been corrected. Please see line 155

**Query 21:**Table 1 : How has the missing data been analysed? included or not included in analysis?

**Response 21:** The individual participants were still included in the analysis. We however note that not all participants had data for all the variables analysed. There were some variables which were missing in some study participants but available in others. We analyzed the data that was available in the study participants; and were able to conduct the Chi-square analyses in STATA. We noted down those that were missing these variables to complete the total number of participants.

**Query 22:** Line 219 - Would suggest WHO Staging instead of WHO Stagingg.

**Response 22:** This has been corrected. Please see line 228

Recommendations:
**Query 23:**1. Would consider further research and analysis into the transferred out, to understand the underlying factors which contribute to the same since it is a large percent of the cohort which was censored.

**Response 23:** Thank you for this comment. Indeed further analysis beyond the scope of this study is required to understand the issue of transfer outs. In our study, the transfer outs were mostly females aged 20-24 years. Further research is required to understand why this population was prone to being transferred out.

**Query 24** :2. Would advocate for the low viral load testing done in your participants as it has significantly reduced the numbers and hence the outcome. To consider ways to boost viral load testing more robustly with use of YCs or an intervention which is deemed fit by you all.

**Response 24:** Thank you for this comment. Further research can certainly look into ways the Youth Champions can boost viral load testing or other interventions that can improve viral load testing in adolescents and youths.

**Query 25:** 3. The full title and short title uses keywords like adolescents which by WHO definition would be just 10-19years, but the median age of ART initiation was 21 years which is more in the bracket of young adult. However there are multiple papers which believe comprehensive adolescent care should include upto 24 years and hence the same must be highlighted in your paper as a need for inclusion of the age group. I think it is important to reflect the same in the title like use of AYLHIV in the title.

**Response 25:** The title and wording has been corrected to reflect that the study population are adolescents and young adults (youths)**.**

Sincerely,

Rachel Chamanga Kanyenda
